# Single Injection of Highly Concentrated Hyaluronic Acid Provides Improvement of Knee Joint Arthrokinematic Motion and Clinical Outcomes in Patients with Osteoarthritis—Non-Randomized Clinical Study

**DOI:** 10.3390/jcm14103557

**Published:** 2025-05-19

**Authors:** Krzysztof Falkowski, Dawid Bączkowicz

**Affiliations:** 1 Department of Orthopaedics and Traumatology of the Musculoskeletal System, Institute of Medical Sciences, University of Opole, 45-758 Opole, Poland; 2 Department of Physiotherapy, Faculty of Physical Education and Physiotherapy, Opole University of Technology, 45-758 Opole, Poland; d.baczkowicz@po.opole.pl

**Keywords:** viscosupplementation, osteoarthritis, vibroarthrography, knee joint

## Abstract

**Background/Objectives**: Intra-articularly administered hyaluronic acid (HA) products improve the mechanical properties of the synovial fluid (SF) in an osteoarthritic (OA) joint and thus improve joint motion quality. However, current diagnostic methods, used to assess the clinical effectiveness of HA-based therapy are based on subjective tools, and are unable to deliver solid data about the actual impact of this molecule on joint functioning. Consequently, the aim of this study was to objectively assess the effect of HA IA injection on joint motion quality with vibroarthrography (VAG) and the subsequent evaluation of patient clinical status. **Methods**: A total of 40 patients with knee OA and 50 healthy individuals as the control group were enrolled in this non-randomized clinical and were subjected to therapy consisting of a single IA administration of highly concentrated HA gel (Biolevox™ HA ONE). The therapy assessment included an evaluation of joint motion quality with the VAG method and a subsequent evaluation of the knee joint function using the WOMAC questionnaire for up to 60 days after the therapy. **Results**: A single IA injection of HA led to an immediate and sustained improvement of the motion quality of OA-affected synovial joints, as proven by the significant reduction in all measured vibroacoustic emissions (VMS, R4, P1, and P2). Furthermore, this was followed by a significant improvement in all WOMAC sub-scales, observed at 30 and 60 days after the therapy. **Conclusions**: The results of this study demonstrate that an IA-HA injection can improve the motion quality of OA-affected joints. Importantly, the observed improvement in joint motion quality is directly correlated with early recovery of joint function. These findings provide objective evidence that HA effectively enhances OA-affected joint biomechanics, contributing to a better understanding of the actual impact of this prevalent OA therapy on knee joint motion quality.

## 1. Introduction

Patients with knee osteoarthritis (OA) represent a highly significant number of people in orthopedic healthcare who require a functioning therapy, as it is estimated that up to 25% of adults experience frequent knee pain related to the chronic process destroying the normal structure of the joint [1,2]. Importantly, due to the progressively aging population combined with the growing obesity problem in developed countries, knee pain is expected to be an increasingly common clinical ailment [3]. The OA of the knee joint is a disease that irreversibly affects all of its compartments (cartilage, synovial membrane, and subchondral bone) by progressive degradation processes. The treatment of OA depends on the stage of progression and includes the use of analgesics and anti-inflammatory drugs; rehabilitation; intra-injections of disease-modifying osteoarthritis drug (DMOADS), corticosteroids, platelet-rich plasma (PRP), hyaluronic acid (HA); and, in advanced stages of the disease, joint replacement [4].

The knee joint is among the most biomechanically complex in the human body, relying on synovial fluid (SF) to minimize friction between articulating cartilage surfaces. SF maintains a consistent layer over cartilage, enabling low-friction movement while also supporting cartilage hydration and nutrient transport. Hyaluronic acid (HA), the primary macromolecular constituent of SF, plays a critical role in determining its viscosity and elasticity. These properties directly influence the fluid’s ability to function as a lubricant and shock absorber. Both the concentration and molecular weight of HA are key determinants of SF’s mechanical performance. With aging and the progression of osteoarthritis (OA), there is a significant reduction in HA content, particularly in terms of molecular weight and overall distribution. This degradation compromises the rheological properties of SF, resulting in its diminished lubrication efficiency and shock absorption, which ultimately impairs joint mobility and mechanical performance [5,6,7].

Additionally, HA fulfills a number of biological and biochemical functions in the synovial joint: (a) it forms a diffusion barrier for plasma cells and large proteins to SF and facilitates the exchange of small substances between synovial capillaries and joint tissues [8]; (b) via interaction with CD44 receptors, HA reduces the production and activity of the pro-inflammatory mediators and extracellular matrix metalloproteinases (MMPs) responsible for cartilage destruction and joint pain [8,9]; (c) HA is able to suppress the expression of collagenase and aggrecanase genes, reducing cartilage degeneration and increasing collagen biosynthesis [10,11]; (d) HA reduces nerve impulses and nerve sensitivity associated with pain, by maintaining the boundary layer around nociceptors [8,12].

Due to the aforementioned mechanical and biological effects of HA, the delivery of exogenous HA via intra-articular (IA) injections (as a therapy called viscosupplementation (VS)) has a deep clinical rationale. It has been shown that IA injected HA has a primarily immediate mechanical effect as it restores the viscoelasticity and lubricating capacity of the synovial fluid, and by doing so, HA reduces friction, finally protecting the cartilage surface from degenerative processes. On top of that, IA injected HA induces long-term biological outcomes like the biosynthesis of endogenous HA and other cartilage extracellular matrix components, which reduces the loss of proteoglycans in cartilage and further chondrocyte apoptosis [12,13].

Currently, the available diagnostic tools, able to evaluate not only the clinical status of the OA-affected joint but also the effectiveness of IA therapies conducted with different HA products, are mostly based on questionnaires and surveys. However, despite the well-proven standardization and well-documented validation, questionnaires like WOMAC, VAS, KOOS, and others are purely subjective tools, highly dependent on the patient’s self-reported feedback, thus being subjected to the patient’s related variability or potential bias [14]. On the other hand, the most commonly used imaging tools, able to objectively evaluate the condition of the OA joint, like radiography, MRI, and CT scans, lack the possibility to assess the direct clinical effect of IA-injected HA. This limitation is based on the fact that IA-injected HA is not able to macroscopically change the structure of the joint, and thus, its effect is not detectable by imaging tools [15].

However, a new, experimental method able to objectively evaluate the joint movement quality has been introduced. Vibroarthrography (VAG) is a diagnostic method is based on the analysis of high-frequency vibroacoustic emissions produced by the mutual movement of cartilaginous surfaces in the synovial joint [16]. The method is based on the phenomenon that under physiological conditions, hyaline cartilage and synovial fluid together create a system with an extremely low coefficient of friction, which directly translates into high motion quality and efficiency. Consequently, since a decrease in cartilage quality and SF mechanical properties is highly related to joint disorders progression, the increase in magnitude of the VAG signal shows a strong correlation with joint pathology progression advancement [17,18,19,20,21]. During OA development and progression, a dramatic decrease in articular cartilage surface quality is observed, which is consistently combined with a profound decrease in the lubrication capacity of the OA-affected SF. The interplay of both leads to increased contact stresses and elevated friction in the joint, which clinically results in impaired motion and can be measured with VAG as increased vibroacoustic emission [20,22,23,24,25]. Importantly, the level of vibroacoustic emission and signal parameters depend on the degree of articular cartilage damage [26]. As a result, VAG signals measured during joint movement can act not only as a very efficient OA development stage diagnostic indicator but can also very efficiently demonstrate the clinical impact of IA-injected agents that affect joint movement quality, e.g., HA. The VAG measurement method is relatively inexpensive, demonstrates high accuracy, sensitivity, and specificity, and is potentially widely available for use in screening conditions [21,25]. The indirect vibration measurement method using accelerometric sensors poses no risk to the test subject and is non-invasive and safe.

The primary aim of this study was to evaluate the immediate clinical effectiveness of a therapy-based single intra-articular (IA) injection of Biolevox™ HA ONE (Biovico, Gdynia, Poland). This was assessed by analyzing the quality of joint motion using the vibroarthrography (VAG) diagnostic method and further validated through assessments of physical function, pain levels, and stiffness improvement following therapy. The secondary aim was to determine whether improvements in joint motion quality are directly associated with the recovery of joint function, thereby supporting the initial clinical effectiveness of the HA-based IA therapy.

## 2. Materials and Methods

The study as a single-center, prospective, unblinded non-randomized clinical trial was registered under the title “Evaluation of knee joint arthrokinematics using vibroacoustic method in patients with gonarthrosis after viscosupplementation”. The study was conducted in accordance with the International Conference on Harmonization (ICH), Good Clinical Practice (GCP) and the World Medical Association’s Declaration of Helsinki. The clinical trial was conducted in accordance with ISO 14155 [27] and in accordance with the Medical Device Directive (93/42/EEC). All procedures used in this study were approved by the Bioethics Committee (no. 242/2019). The principal investigator obtained written informed consent from all patients prior to the start of the procedures related to the study. The research started in November 2019.

### 2.1. Patients

The study included 40 patients who met the inclusion criteria, of which 7 patients did not participate in further follow-up or did not wish to complete the study. The control group consisted of 50 asymptomatic subjects with similar anthropometric parameters. The disease progression in the knee joint of the included patients were assessed according to the Kellgren–Lawrence (K-L) scale on the basis of an X-ray. Patients with knee and bone injuries with damage to the articular surface and diseases other than OA were excluded from the study. Patient inclusion and exclusion criteria, used to minimize the potential of bias induced due to nonrandomization, are summarized in Table 1. The participants of this clinical trial were recruited from the patients of Department of Trauma and Orthopaedic Surgery at University Clinical Hospital in Opole. A sample-size determination analysis is described in Appendix A; additionally, the flow-chart of patient selection during the study is presented as Figure S1.

### 2.2. Product Description

The product used in this study was Biolevox™ HA ONE (Biovico Sp. z o.o., Gdynia, Poland), a 2.5% sodium hyaluronate solution in the form of a viscoelastic gel dedicated for VS of synovial joints. Biolevox™ HA ONE contains 120 mg of HA in the 4.8 mL pre-filled sterile syringe. Biolevox™ HA ONE has one of the highest concentrations of HA, and it is recommended for single-injection therapy.

### 2.3. Trial Design and Clinical Assessment

The movement quality was assessed indirectly using accelerometric sensors that record the vibroacoustic signal produced by joint structures during joint movement. VAG data were recorded with sensor model 4508B-001 (Brüel & Kjær Sound & Vibration Measurement A/S, Nærum, Denmark), and further amplified with signal amplifier type 1704-A-002 CCLD (Brüel & Kjær Sound & Vibration Measurement A/S, Nærum, Denmark). The test took place in a sitting position with a sensor attached to the skin 1 cm above the patella and consisted of an alternating motion of straightening and flexion of the test knee in the range of 90°–0°–90° in an open kinematic chain. Detailed descriptions of the VAG method are given in Supplementary Materials. The vibroacoustic signal assessment included 5 measurements: (1) prior to HA injection; (2) immediately after HA injection; (3) 7 days after the HA injection; (4) 30 days after the HA injection; (5) 60 days after the HA injection.

In addition, patient functional status was assessed using the Western Ontario and McMaster Universities Osteoarthritis Index (WOMAC) questionnaire before the HA administration and 7, 30, and 60 days after the injection.

The control group included in this trial was tested once with an accelerometer sensor, which allow the presentation of the VAG results of the non OA-affected population. A detailed description of study intervention regimen is provided in Supplementary Materials.

To determine the safety of therapy based on IA injection of Biolevox™ HA ONE, participants were monitored for adverse events (AEs) and serious adverse events (SAEs).

The primary effectiveness outcome measure of the study was the improvement in the WOMAC total score from baseline to the 2-month follow-up visit. Secondary endpoints were as follows: (i) evaluation of the therapy effectiveness according VAG measurements; (ii) safety of the investigational medical device.

### 2.4. Statistical Analysis

Data were expressed as a box and whiskers plots with min to max values. Values with a *p* < 0.05 were considered to be statistically significant. Statistical differences for the results of VAG values (VMS, R4, P1, and P2) and WOMAC scales were calculated with Friedman and Kruskal–Wallis tests with Dunn’s multiple comparison test, as well as with a repeated-measures one-way ANOVA with Tukey multiple comparison test. A correlation analysis was performed with use of the Spearman test. Data were analyzed and presented with use of the Prism GraphPad ver.9.0 software package.

## 3. Results

**Figure 1:**
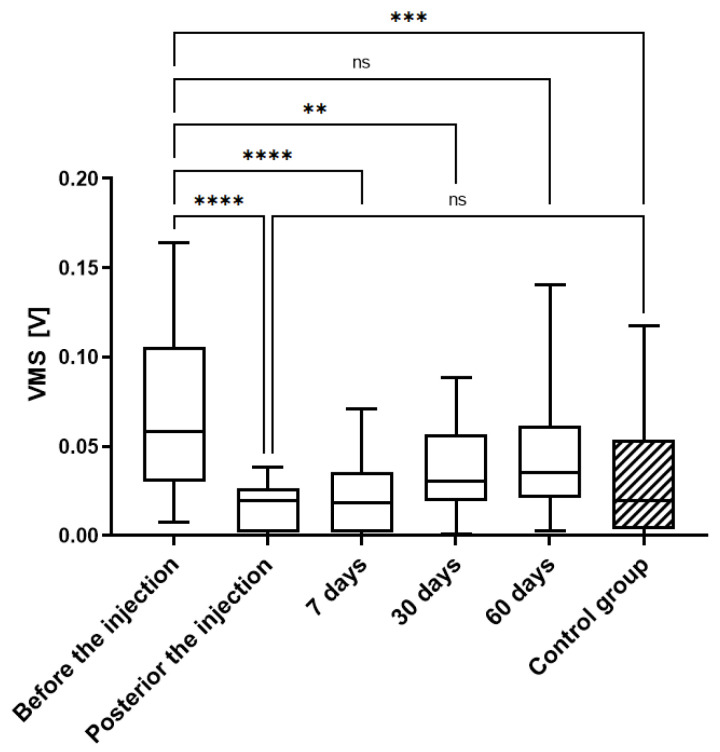
Variation of mean square (VMS) parameter values in analyzed research groups. Applied intra-articular injection caused immediate, statistically significant decrease in analyzed parameter. Over period of follow-up time points, a negligible increase in VMS was observed. Importantly, the analysis showed non-significant difference between control group and patients after injection; ns—non-significant; **—*p* < 0.01; ***—*p* < 0.001; ****—*p* < 0.0001.

**Figure 2:**
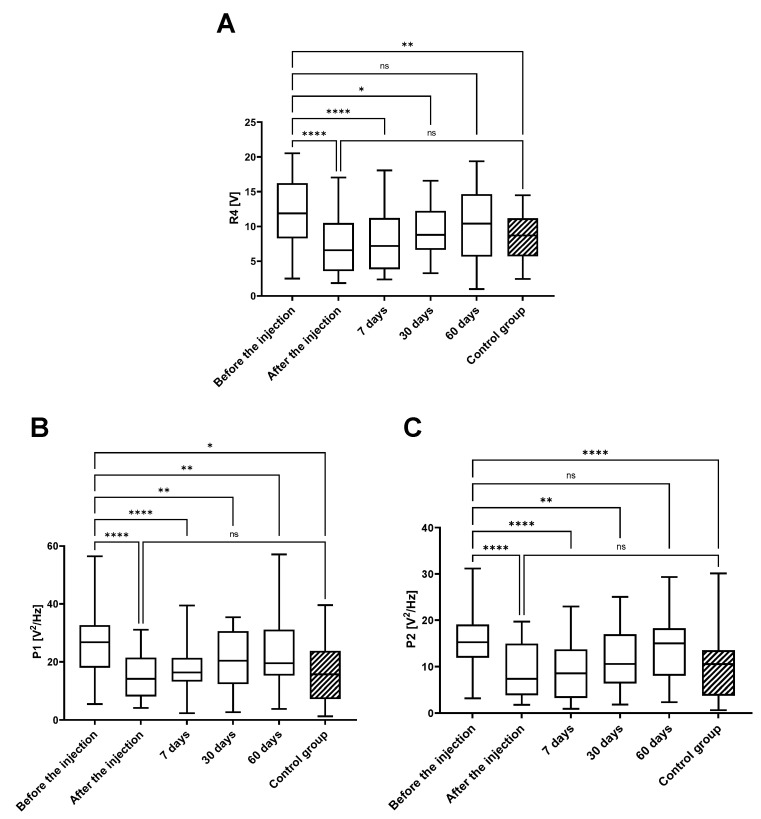
Values of R4 (**A**), P1 (**B**), and P2 (**C**) parameters in analyzed research groups. The introduced viscosupplementation led to an immediate and statistically significant reduction in the analyzed parameters, followed by a minimal rise over the course of the follow-up points; ns—non-significant; *—*p* < 0.05; **—*p* < 0.01; ****—*p* < 0.0001.

The demographic data are presented in Table S1, with no significant differences between the analyzed variables. To assess the quality of the arthrokinematic motion of the joint, the VAG parameters were analyzed based on measurements at five time points for the treated-participant group and once for asymptomatic-volunteer control group (Figure 1 and Figure 2 and Table S2).

For all VAG parameters, the results obtained before the therapy were nearly 2-fold higher when compared to VAG parameters in the control group, and this was characterized by the significant statistical difference, for P2 (*p* < 0.0001), VMS (*p* < 0.001), P4 (*p* < 0.01), and the P1 parameter (*p* < 0.05).

A further analysis showed that the values of all the VAG parameters were significantly reduced immediately after the VS, highlighted by the high statistical differences (*p* < 0.0001). The IA administration of HA resulted in a decrease in VMS, P1, P2, and R4 parameters to the level of the control (healthy subjects). At the first point of follow-up, 7 days after VS, the significant differences (*p* < 0.0001) were still observed in all four VAG parameters when compared to levels before the injection. Subsequently, this reduction could still be observed at 30 days post-HA injection, as all analyzed VMS values were statistically reduced when compared to levels prior to HA injection and all reductions were statistically significant: *p* < 0.01, *p* < 0.01, *p* < 0.01, and *p* < 0.05, for VMS, P1, P2, and R4, respectively. At 60 days post-injection, the previously reduced levels of VMS, P2, and R4 parameter values increased and were not significantly reduced when compared to levels prior to injection. The only exception was the power spectral density for frequencies of 50 to 250 Hz (P1), which maintained significantly lower levels (*p* < 0.01).

**Figure 3:**
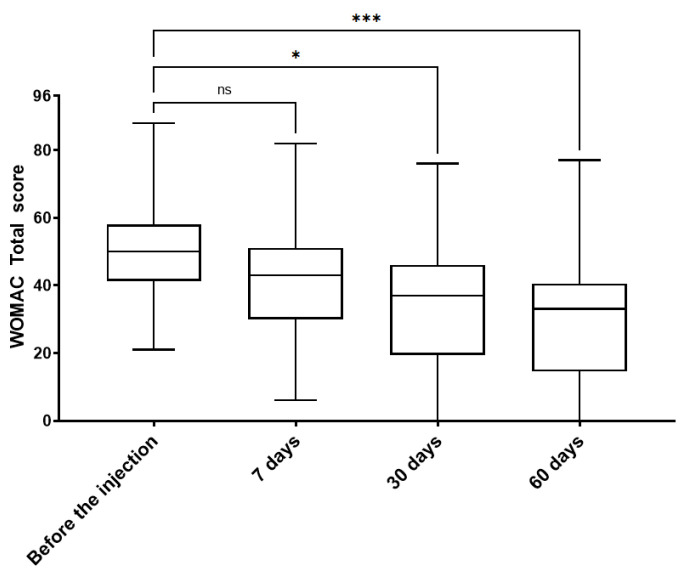
WOMAC total score of patients receiving viscosupplementation. Treatment of intra-articular injection caused stable improvement of analyzed score through follow-up points, with the most significant difference at 60 days post-treatment; ns—non-significant; *—*p* < 0.05; ***—*p* < 0.001.

**Figure 4:**
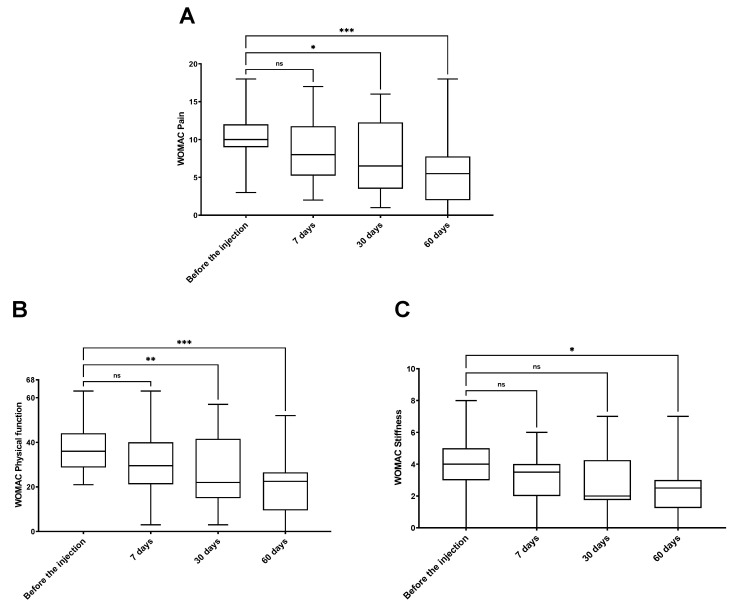
WOMAC pain (**A**), physical function (**B**) and stiffness (**C**) subscales in analyzed research groups. The implemented treatment was significantly efficient in improvement of analyzed WOMAC subscales, providing pain relief and functional improvement; ns—non-significant; *—*p* < 0.05; **—*p* < 0.01; ***—*p* < 0.001.

In order to monitor patients’ functional status across the study, all enrolled patients were assessed by WOMAC questionnaire prior to therapy and at all four follow-up time points (Figure 3 and Figure 4 and Table S2).

The WOMAC total score showed no significant differences after 7 days post-injection when compared to the values before HA administration. Nevertheless, already at the second follow-up time point (30 days after the VS), the WOMAC total score showed a significant decrease (*p* < 0.05). This was followed by an even further decrease at the third follow-up point—60 days after the injection (*p* < 0.001) (Figure 3). A further analysis of the WOMAC subscales- WOMAC pain, physical function, and stiffness confirmed the improvement of patient functional status. The values of WOMAC pain and physical function scales were significantly decreased at 30 days after VS (*p* < 0.05 and *p* < 0.01, respectively). Later, at 60 days after the therapy, an even further and more significant reduction was observed in all WOMAC subscales values. Patients declared improvements in pain (*p* < 0.001), physical function (*p* < 0.001), and stiffness (*p* < 0.05). The results of all the WOMAC sub-scales show a stable improvement of all subscale values could be observed through all the follow-up points (Figure 4).

**Figure 5:**
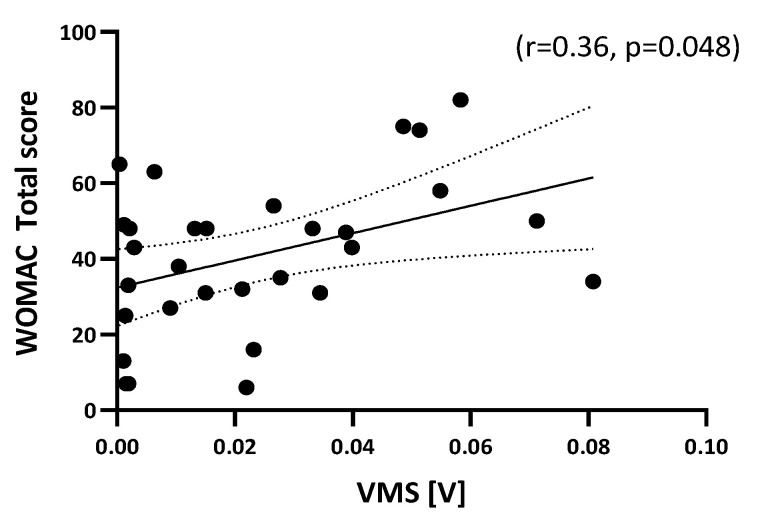
Correlation analysis with linear regression of WOMAC total score and VMS parameter 7 days after treatment. Regression analysis (solid line) with 95% CI (dotted line) depicted.

In order to investigate if the improvement of VAG parameters could be related to the improvement of patient functional status, we analyzed the correlation of different VAG parameters and WOMAC scores across different follow-up time points. From all conducted correlation analyses, a statistically significant correlation, particularly between WOMAC total score and VMS parameter at 7 days after HA-based therapy, was noted (*p* < 0.05) (Figure 5 and Figure S1).

Safety monitoring showed no adverse events related to the IA administration of the HA-based product during the whole duration of the study. No case of a septic complication and no serious adverse events (SAEs) were reported.

## 4. Discussion

IA-HA injections have been shown by numerous independent studies to be clinically effective in reducing pain and improving the overall clinical functionality of OA patients [28]. Nevertheless, individual reports indicate considerable variability in clinical response and the subsequent long-term benefit of therapy based on distinct HA-based products. These differences have been attributed to specific properties of individual HA products, such as molecular weight (MW), HA concentration, HA source, and the recommended number of injections per treatment cycle. For instance, therapies utilizing HA with an optimally high MW and elevated HA concentration are posited to more effectively enhance the rheological properties of osteoarthritis-affected synovial fluid (SF) [29], thereby suggesting a prolonged clinical benefit [13].

Conversely, products characterized by a low HA concentration and low MW often necessitate multiple intra-articular (IA) injections within a single treatment cycle, and their clinical efficacy remains highly debatable or even negligible [30]. HA product parameters have been demonstrated to significantly influence the duration of clinical effects, with certain products showing efficacy for up to six months [31,32,33,34,35,36], while others are reported to maintain their effectiveness for a year or longer [37,38,39]. Notably, products with a low HA concentration yet high MW are still able to provide clinical effectiveness for up to 6 months [40]. These discrepancies in key HA product parameters, along with variations in products quality, contribute to huge inconsistencies within the field of viscosupplementation. The market is flooded with various HA-based products, spanning from those of high quality to others of markedly inferior quality. This leads to a situation where the overall judgment about the clinical functionality of HA intra-articular injections—and, by extension, viscosupplementation as a therapy for OA—is based on generalized conclusions and meta-analyses based on imprecise inclusion criteria. Moreover, these generalized conclusions have been adopted by numerous prestigious societies as the foundation for their guidelines, which question the clinical rationale for using viscosupplementation in OA [41].

Nevertheless, the validity of conclusions regarding the potential lack of clinical functionality of HA-based therapy in OA remains highly debatable, especially when they are based only on subjective evaluations such as VAS, WOMAC, and other questionnaires. Especially in disease like OA, where the pain factor plays such a significant role, and thus the placebo effect is actually the main decisive factor, albeit one entirely dependent on the patient’s related variability and potential bias [42].

Consequently, in our opinion, supplementing the questionnaire data with objective clinical assessment techniques and measurement methods, such as VAG, independently of patient-specific factors will be able to provide reliable data on the actual effectiveness of HA-based therapy in OA. Moreover, using the VAG measurement technique, it has been demonstrated that IA injections of HA are effective in improving joint arthrokinematic properties, reducing friction, and enhancing joint motion quality in patients with OA [22,43]. In a study published by Bączkowicz et al. (2021) [22], similarly to the present one, before receiving HA injections, OA patients exhibited VAG parameter values nearly twice as high as those of healthy controls, indicating a significant diminishment in joint movement quality, associated with disease stage or advancement. Subsequently, two weeks after the IA-HA injection, the particular values of VAG measurement were significantly decreased when compared to the pre-injection status. Nevertheless, a relatively short duration of improvement in joint motion quality measured with VAG was achieved, as 4 weeks after the injection, the measured VAG parameters did not differ significantly from the values obtained before the HA injection [22]. This remains in contrast with results obtained in our study, as we noticed that even 30 days (more than 4 weeks) after the HA injection, all measured VAG values were still significantly reduced when compared to values prior to the HA injection. Moreover, in the present study, one particular VAG measurement parameter (P1) was shown to still be significantly reduced even 60 days after the HA injection. These differences in the duration of HA’s beneficial effect on joint motion quality measured with VAG suggest that the parameters of HA used in this study are most likely better suited to function as motion-quality-improving agents in OA. Interestingly, however, both HA-based products used by Bączkowicz [22] and in our study were of high molecular weight and were used as single-shot products. The major difference in both products was primarily in the HA concentration, which determined the total amount of HA in the therapy. The product used by Bączkowicz et al. (2021) [22] had a relatively low HA concentration (1.5%), and thus, the therapy delivered only 30 mg of HA in a 2 mL injection. The HA used in our study had a very high concentration (2.5%), and thus, the therapy delivered 120 mg of HA in a 4.8 mL injection. This is four times more HA in the therapy that was able to facilitate the OA-affected SF properties, improving its viscosity and enhancing its ability to lubricate the cartilage. This most likely resulted in a prolonged improvement in joint motion quality, measured with VAG. Indeed, the importance of using hyaluronic acid (HA) with an appropriate molecular weight (MW) in intra-articular (IA) therapy for osteoarthritis (OA) has been demonstrated by Altman et al. (2016) [44]. The authors showed that only HA with a selectively high molecular weight is capable of eliciting the expected therapeutic response. Notably, these findings were subsequently confirmed by other studies, further reinforcing the significant role of sufficiently high MW HA in achieving an effective OA treatment [45]. Notably, this phenomenon may be partially attributed to the fact that only HA with an optimally high MW can effectively signal through the HA-specific CD44 receptor, thereby initiating a meaningful biological response that translates into a clinical response [46,47].

Cross-linked HAs, as those with an exceptionally high MW, have demonstrated high resistance to enzymatic degradation, allowing them to remain longer within the synovial joint environment [48]. Based on this characteristic, it is commonly assumed that cross-linked HAs should offer prolonged improvement in joint movement quality in osteoarthritis (OA)-affected joints. This assumption has contributed to a widespread belief that cross-linked HAs exert longer-lasting clinical effects following intra-articular (IA) injection. However, this hypothesis lacks conclusive evidence. In fact, the clinical effectiveness of cross-linked HAs typically does not exceed six months—comparable to that of non-cross-linked HAs with optimized parameters [49]. Furthermore, the presumed extended benefit of cross-linked HAs has been directly challenged in a study by Bączkowicz et al. (2021) [22], which reported that cross-linked HAs improved joint movement quality for no more than two weeks. These findings suggest that low-concentration cross-linked HAs may provide less sustained improvement than linear, highly concentrated HAs such as those utilized in our study.

It is generally believed that, in the long term, restoring proper mobility and movement quality in an OA joint through IA-HA injection can help re-establish the joint’s metabolic and rheological homeostasis. This restoration is expected to lead to sustained biological effects and corresponding clinical benefits, including pain reduction and improved joint function [50]. This was confirmed by our study, as we demonstrated a significant correlation between reduction in vibroacoustic emission and improvement in joint functionality score with WOMAC at 7 days after HA injection. This finding strongly suggests that the recovery of joint function in OA-affected joints can be directly attributed to the restoration of proper joint motion quality facilitated by the injected HA. Consequently, the time response shift between improvement in joint movement quality and restoration of joint function, we hypothesize that VS as a therapy might have a two-phase effect on the OA-affected joint condition. Initially, VS induces an immediate mechanical response, likely due to the restoration of synovial fluid rheological properties, which leads to improved movement quality. This early mechanical improvement serves as a precursor to subsequent biological responses, ultimately resulting in longer-term clinical benefits [51].

The authors of this study are aware that the conducted study has its limitations. Firstly, the patient follow-up time was only 60 days, and this period did not allow a conclusion to be drawn regarding the long-lasting clinical effect of the HA used in this study, and thus confirm that chosen product properties are able to provide a highly sustained clinical effect. Nonetheless, the observation period was adequate to assess the medium-term impact of viscosupplementation (VS) on knee arthrokinematics and to draw meaningful conclusions regarding the role of improved joint movement quality in subsequent clinical outcomes. Second, although a healthy control group was included, the study lacked a direct experimental control group.

It is clear that the inclusion of a direct experimental injection control—such as saline or a hyaluronic acid with different physicochemical properties—would have strengthened the study design. This would have allowed for a more precise assessment of the extent to which the clinical effectiveness of the tested HA is attributable to its ability to improve joint movement quality. Nevertheless, as we mentioned in the Introduction, the HA market offers a wide variety of products, making it difficult to choose the most suitable one for the control. Clearly, it would be questionable why a particular product was included as a control group and why others were not.

On the other hand, including a saline as a control group is considered unethical, and it is not accepted by ethical committees.

Despite this, the inclusion of VAG measurements from a healthy control group confirmed the validity of the vibroacoustic method as a reliable diagnostic tool and supported the conclusion that the HA was effective in improving joint movement quality.

## 5. Conclusions

Firstly, the results of this research confirmed with fully objective vibroacoustic measurements that the IA injection of highly concentrated HA characterized with an optimally high molecular weight enables the full restoration of correct joint movement quality. This effect is achieved by enhancing the lubricating properties of the synovial fluid and reducing friction between joint surfaces. Secondly, the study establishes a direct link between improvements in joint motion quality and the subsequent clinical effectiveness of therapy using intra-articular HA with highly specified physicochemical properties. Finally, this is the first study to show that such long-term improvements in joint movement quality—and the resulting clinical benefits—can only be achieved with HA formulations characterized by a high concentration, thereby delivering a substantial amount of hyaluronate during treatment.

## Figures and Tables

**Table 1:** Screening criteria of enrolled patients.

**Inclusion Criteria**	**Exclusion Criteria**
‑ Level II and III of degenerative changes in knee joint based on K-L scale; ‑ Obtaining more than 20 points in WOMAC scale; ‑ Preserved mobility of the knee joint in a minimum range of 0 (full extension)–90 degrees (flexion); ‑ Qualification for viscosupplementation with hyaluronic acid.	Intra-articular injections of steroids or other injectables up to one year before the study; History of knee joint trauma during last 2 years or any trauma with articular cartilage-related damage; Occurrence of knee joint dysfunctions other than osteoarthritis, including symptomatic arthritis.

## Data Availability

All additional data are contained in the Supplementary Materials.

## Supplementary Materials

The following supporting information can be downloaded at: https://www.mdpi.com/article/10.3390/jcm14103557/s1, Table S1. Demographic characteristics of patients included in the study. Table S2. Mean values with standard deviation of vibroarthography parameters and WOMAC questionnaire score in analyzed groups. Figure S1. Flow-chart of patient selection during the study.

## Appendix A

The number of patients included in this study was determined based on WOMAC total scale data (primary endpoint). The sample size was calculated based on the standard sample size counting methods for a dependent data, with the assumption that the clinically relevant improvement of the analyzing scale in the knee joint as a result of viscosupplementation is 20% or more. The calculations included a 20% dropout rate from the study. The sample size was based on a power of 80%. A two-sided *p* value *p* < 0.05 was considered statistically significant. The sample size calculation showed that in order to ensure the 99% confidence with a power of 80% (two-sided testing), a sample size of 32 participants was needed.

## References

[1] Nguyen U.-S.D.T., Zhang Y., Zhu Y., Niu J., Zhang B., Felson D.T. (2011). Increasing Prevalence of Knee Pain and Symptomatic Knee Osteoarthritis: Survey and Cohort Data. Ann. Intern. Med..

[2] Turkiewicz A., Gerhardsson De Verdier M., Engstrom G., Nilsson P.M., Mellstrom C., Lohmander L.S., Englund M. (2015). Prevalence of Knee Pain and Knee OA in Southern Sweden and the Proportion That Seeks Medical Care. Rheumatology.

[3] Turkiewicz A., Petersson I.F., Björk J., Dahlberg L.E., Englund M. (2013). The Consultation Prevalence of Osteoarthritis 2030 May Increase by 50%: Prognosis for Sweden. Osteoarthr. Cartil..

[4] Bird H.A. (2003). Controversies in the Treatment of Osteoarthritis. Clin. Rheumatol..

[5] Schurz J., Ribitsch V. (1987). Biorheology of Synovial Fluid1. BIR.

[6] Sabaratnam S., Arunan V., Coleman P.J., Mason R.M., Levick J.R. (2005). Size Selectivity of Hyaluronan Molecular Sieving by Extracellular Matrix in Rabbit Synovial Joints. J. Physiol..

[7] Hui A.Y., McCarty W.J., Masuda K., Firestein G.S., Sah R.L. (2012). A Systems Biology Approach to Synovial Joint Lubrication in Health, Injury, and Disease. Wiley Interdiscip. Rev. Syst. Biol. Med..

[8] Ghosh P., Guidolin D. (2002). Potential Mechanism of Action of Intra-Articular Hyaluronan Therapy in Osteoarthritis: Are the Effects Molecular Weight Dependent?. Semin. Arthritis Rheum..

[9] Waddell D.D., Kolomytkin O.V., Dunn S., Marino A.A. (2007). Hyaluronan Suppresses IL-1β-Induced Metalloproteinase Activity from Synovial Tissue. Clin. Orthop. Relat. Res..

[10] Altman R.D., Dasa V., Takeuchi J. (2018). Review of the Mechanism of Action for Supartz FX in Knee Osteoarthritis. CARTILAGE.

[11] Karna E., Miltyk W., Pałka J.A., Jarząbek K., Wołczyński S. (2006). Hyaluronic Acid Counteracts Interleukin-1-Induced Inhibition of Collagen Biosynthesis in Cultured Human Chondrocytes. Pharmacol. Res..

[12] Forsey R., Fisher J., Thompson J., Stone M., Bell C., Ingham E. (2006). The Effect of Hyaluronic Acid and Phospholipid Based Lubricants on Friction within a Human Cartilage Damage Model. Biomaterials.

[13] Moreland L.W. (2003). Intra-Articular Hyaluronan (Hyaluronic Acid) and Hylans for the Treatment of Osteoarthritis: Mechanisms of Action. Arthritis Res. Ther..

[14] McConnell S., Kolopack P., Davis A.M. (2001). The Western Ontario and McMaster Universities Osteoarthritis Index (WOMAC): A Review of Its Utility and Measurement Properties. Arthritis Rheum..

[15] Patel R., Orfanos G., Gibson W., Banks T., Mcconaghie G., Banerjee R. (2024). Viscosupplementation with High Molecular Weight Hyaluronic Acid for Hip Osteoarthritis: A Systematic Review and Meta-Analysis of Randomised Control Trials of the Efficacy on Pain, Functional Disability, and the Occurrence of Adverse Events. Acta Chir. Orthop. Traumatol. Cech..

[16] Łysiak A., Froń A., Bączkowicz D., Szmajda M. (2020). Vibroarthrographic Signal Spectral Features in 5-Class Knee Joint Classification. Sensors.

[17] Bączkowicz D., Majorczyk E., Kręcisz K. (2015). Age-Related Impairment of Quality of Joint Motion in Vibroarthrographic Signal Analysis. BioMed Res. Int..

[18] Kręcisz K., Bączkowicz D., Kawala-Sterniuk A. (2022). Using Nonlinear Vibroartrographic Parameters for Age-Related Changes Assessment in Knee Arthrokinematics. Sensors.

[19] Karpiński R., Krakowski P., Jonak J., Machrowska A., Maciejewski M., Nogalski A. (2021). Estimation of Differences in Selected Indices of Vibroacoustic Signals between Healthy and Osteoarthritic Patellofemoral Joints as a Potential Non-Invasive Diagnostic Tool. J. Phys.: Conf. Ser..

[20] Bączkowicz D., Kręcisz K., Borysiuk Z. (2019). Analysis of Patellofemoral Arthrokinematic Motion Quality in Open and Closed Kinetic Chains Using Vibroarthrography. BMC Musculoskelet. Disord..

[21] Kręcisz K., Bączkowicz D. (2018). Analysis and Multiclass Classification of Pathological Knee Joints Using Vibroarthrographic Signals. Comput. Methods Programs Biomed..

[22] Bączkowicz D., Skiba G., Szmajda M., Vařeka I., Falkowski K., Laudner K. (2021). Effects of Viscosupplementation on Quality of Knee Joint Arthrokinematic Motion Analyzed by Vibroarthrography. Cartilage.

[23] Bączkowicz D., Kręcisz K. (2013). Vibroarthrography in the Evaluation of Musculoskeletal System—A Pilot Study. Ortop. Traumatol. Rehabil..

[24] Bączkowicz D., Majorczyk E. (2014). Joint Motion Quality in Vibroacoustic Signal Analysis for Patients with Patellofemoral Joint Disorders. BMC Musculoskelet. Disord..

[25] Tanaka N., Hoshiyama M. (2012). Vibroarthrography in Patients with Knee Arthropathy. J. Back. Musculoskelet. Rehabil..

[26] Bączkowicz D., Majorczyk E. (2016). Joint Motion Quality in Chondromalacia Progression Assessed by Vibroacoustic Signal Analysis. PMR.

[27] (2018). Clinical Investigation of Medical Devices for Human Subjects—Good Clinical Practice. https://www.iso.org/standard/71690.html.

[28] Bannuru R.R., Natov N.S., Dasi U.R., Schmid C.H., McAlindon T.E. (2011). Therapeutic Trajectory Following Intra-Articular Hyaluronic Acid Injection in Knee Osteoarthritis—Meta-Analysis. Osteoarthr. Cartil..

[29] Mensitieri M., Ambrosio L., Iannace S., Nicolais L., Perbellini A. (1995). Viscoelastic Evaluation of Different Knee Osteoarthritis Therapies. J. Mater. Sci: Mater. Med..

[30] Arrich J. (2005). Intra-Articular Hyaluronic Acid for the Treatment of Osteoarthritis of the Knee: Systematic Review and Meta-Analysis. Can. Med. Assoc. J..

[31] Petrella R.J., DiSilvestro M.D., Hildebrand C. (2002). Effects of Hyaluronate Sodium on Pain and Physical Functioning in Osteoarthritis of the Knee: A Randomized, Double-Blind, Placebo-Controlled Clinical Trial. Arch. Intern. Med..

[32] Miltner O., Schneider U., Siebert C.H., Niedhart C., Niethard F.U. (2002). Efficacy of Intraarticular Hyaluronic Acid in Patients with Osteoarthritis—a Prospective Clinical Trial. Osteoarthr. Cartil..

[33] Frizziero L., Govoni E., Bacchini P. (1998). Intra-Articular Hyaluronic Acid in the Treatment of Osteoarthritis of the Knee: Clinical and Morphological Study. Clin. Exp. Rheumatol..

[34] Altman R.D., Moskowitz R. (2000). Intra-Articular Sodium Hyaluronate Reduces Pain and Improves Function in Osteoarthritis of Knee. J. Rheumatol..

[35] Huang T.-L., Chang C.-C., Lee C.-H., Chen S.-C., Lai C.-H., Tsai C.-L. (2011). Intra-Articular Injections of Sodium Hyaluronate (Hyalgan®) in Osteoarthritis of the Knee. a Randomized, Controlled, Double-Blind, Multicenter Trial in the Asian Population. BMC Musculoskelet. Disord..

[36] Huskisson E. (1999). Hyaluronic Acid in the Treatment of Osteoarthritis of the Knee. Rheumatology.

[37] Gimeno Del Sol M., Trueba Davalillo C.Á., Trueba Vasavilbaso C., Navarrete Álvarez J.M., Coronel Granado M.P., García Jiménez O.A., Gil Orbezo F. (2015). Clinical Efficacy of Intra-Articular Injections in Knee Osteoarthritis: A Prospective Randomized Study Comparing Hyaluronic Acid and Betamethasone. Open Access Rheumatol. Res. Rev..

[38] Neustadt D.H. (2003). Long-Term Efficacy and Safety of Intra-Articular Sodium Hyaluronate (Hyalgan) in Patients with Osteoarthritis of the Knee. Clin. Exp. Rheumatol..

[39] Kolarz G., Kotz R., Hochmayer I. (2003). Long-Term Benefits and Repeated Treatment Cycles of Intra-Articular Sodium Hyaluronate (Hyalgan) in Patients with Osteoarthritis of the Knee. Semin. Arthritis Rheum..

[40] Farì G., Mancini R., Dell’Anna L., Ricci V., Della Tommasa S., Bianchi F.P., Ladisa I., De Serio C., Fiore S., Donati D. (2024). Medial or Lateral, That Is the Question: A Retrospective Study to Compare Two Injection Techniques in the Treatment of Knee Osteoarthritis Pain with Hyaluronic Acid. J. Clin. Med..

[41] Brophy R.H., Fillingham Y.A. (2022). AAOS Clinical Practice Guideline Summary: Management of Osteoarthritis of the Knee (Nonarthroplasty), Third Edition. J. Am. Acad. Orthop. Surg..

[42] Neogi T., Colloca L. (2023). Placebo Effects in Osteoarthritis: Implications for Treatment and Drug Development. Nat. Rev. Rheumatol..

[43] Falkowski K., Skiba G., Czerner M., Szmajda M., Bączkowicz D. (2018). Effects of Viscosupplementation on Knee Joint Arthrokinematics—Pilot Study. Ortop. Traumatol. Rehabil..

[44] Altman R.D., Bedi A., Karlsson J., Sancheti P., Schemitsch E. (2016). Product Differences in Intra-Articular Hyaluronic Acids for Osteoarthritis of the Knee. Am. J. Sports Med..

[45] Migliorini F., Maffulli N., Nijboer C.H., Pappalardo G., Pasurka M., Betsch M., Kubach J. (2025). Comparison of Different Molecular Weights of Intra-Articular Hyaluronic Acid Injections for Knee Osteoarthritis: A Level I Bayesian Network Meta-Analysis. Biomedicines.

[46] Wang C.-T., Lin Y.-T., Chiang B.-L., Lin Y.-H., Hou S.-M. (2006). High Molecular Weight Hyaluronic Acid Down-Regulates the Gene Expression of Osteoarthritis-Associated Cytokines and Enzymes in Fibroblast-like Synoviocytes from Patients with Early Osteoarthritis. Osteoarthr. Cartil..

[47] Smith M.M., Ghosh P. (1987). The Synthesis of Hyaluronic Acid by Human Synovial Fibroblasts Is Influenced by the Nature of the Hyaluronate in the Extracellular Environment. Rheumatol. Int..

[48] Edsman K., Hjelm R., Lärkner H., Nord L.I., Karlsson A., Wiebensjö Å., Höglund A.U., Kenne A.H., Näsström J. (2011). Intra-Articular Duration of Durolane^TM^ after Single Injection into the Rabbit Knee. Cartilage.

[49] McGrath A.F., McGrath A.M., Jessop Z.M., Gandham S., Datta G., Dawson-Bowling S., Cannon S.R. (2013). A Comparison of Intra-Articular Hyaluronic Acid Competitors in the Treatment of Mild to Moderate Knee Osteoarthritis. J. Arthritis.

[50] Bellamy N., Campbell J., Welch V., Gee T.L., Bourne R., Wells G.A. (2006). Viscosupplementation for the Treatment of Osteoarthritis of the Knee. Cochrane Database Syst. Rev..

[51] Bowden D.J., Byrne C.A., Alkhayat A., Eustace S.J., Kavanagh E.C. (2017). Injectable Viscoelastic Supplements: A Review for Radiologists. Am. J. Roentgenol..

[52] Falkowski K., Madej W., Hubeny J. (2024). Single Intra-Articular Injection of High-Molecular and Highly Concentrated Hyaluronic Acid Improves Arthrokinematics in the Knee Joint, Resulting in a Significant Clinical Outcome. Osteoarthr. Cartil..

