# Supplementary material for: Single Injection of Highly Concentrated Hyaluronic Acid Provides Improvement of Knee Joint Arthrokinematic Motion and Clinical Outcomes in Patients with Osteoarthritis—Non-Randomized Clinical Study
Source: J Clin Med. 2025 May 19;14(10):3557. doi: 10.3390/jcm14103557 (PMC12112193; doi:10.3390/jcm14103557)
Supplement: Supplementary file 1 [file jcm-14-03557-s001.zip › jcm-3563737-supplementary.pdf]

Supplementary materials

Single injection of highly concentrated hyaluronic acid provides improvement of knee joint arthrokinematic motion and clinical outcomes in patients with osteoarthritis – non-randomized clinical study.

Table S1. Demographic characteristics of patients included in the study.

		Treated group (n=33)		Control group (n=50)	
		Mean (SD)	n (%)	Mean (SD)	n (%)
Age		57.8 (6.1)		56.2 (5.4)	
Gender	Female		29 (87.9)		42 (84.0)
	Male		4 (12.1)		8 (16.0)
Height (cm)		165.3 (7.5)		162.6 (6.8)	
Weight (kg)		73.4 (4.2)		71.6 (3.8)	
BMI (kg/cm2)		26.2 (4.1)		25.9 (3.3)	
Duration of OA (years)		6.9 (4.2)		N/A	

Table S2. Mean values with standard deviation of vibroarthography parameters and WOMAC questionnaire score in analyzed groups.

		Analyzed research groups					
		Prior HA injection	After HA injection	7 days follow-up	30 days follow-up	60 days follow-up	Control group
VAG parameters	VMS	0.067 (0.048)	0.017 (0.012)	0.022 (0.022)	0.035 (0.025)	0.043 (0.036)	0.037 (0.036)
	P1	26.98 (12.51)	15.25 (7.79)	16.64 (8.75)	19.96 (9.53)	22.64 (11.61)	15.84 (9.82)
	P2	15.57 (6.53)	9.08 (5.94)	9.24 (6.41)	11.62 (6.52)	13.99 (6.82)	10.27 (6.56)
	R4	12.34 (4.80)	7.27 (4.36)	7.73 (4.30)	9.08 (3.57)	10.21 (4.97)	8.60 (3.26)
WOMAC questionnaire	WOMAC Total	50.53 (16.50)		41.00 (20.06)	35.52 (21.56)	30.67 (20.15)	
	WOMAC Pain	10.43 (3.58)		8.25 (4.04)	7.32 (4.60)	5.63 (4.67)	
	WOMAC Physical function	37.89 (10.71)		30.11 (15.09)	26.59 (16.32)	20.81 (14.85)	
	WOMAC Stiffness	4.00 (2.04)		3.18 (1.61)	2.91 (2.25)	2.56 (1.97)	

* HA – hyaluronic acid; VAG – vibroarthography

Material and methods

Trial design and clinical assessment

A vibration measuring sensor model 4508B-001 (Brüel & Kjær Sound & Vibration Measurement A/S, Nærum, Denmark), a signal amplifier type 1704-A-002 CCLD (Brüel & Kjær Sound & Vibration Measurement A/S, Nærum, Denmark) and an analog-to-digital converter, connected to a PC, were used for the VAG test. The sensors were attached to the skin using double-sided adhesive tape 1 cm above the top of the patella. Once the sensors were attached, the subject performed movement at the knee joint according to the metronome's timing of 82 beats per minute. The test took place in a sitting position and consisted of an alternating motion of straightening and flexion of the test knee in the range of 90°-0°-90° in an open kinematic chain. The expected duration of the test was about 6 seconds, during which 4 complete cycles of straightening/flexion were performed each. The VAG parameters (variation of mean square (VMS), average of the four minimum and maximum values (R4), and power spectral density for frequency of 50 to 250 Hz (P1), and 250 to 450 Hz (P2)) were measured in the study, reflecting the level of vibration generated during movement.

The vibroacoustic signal assessment included 5 measurements: 1) prior HA injection; 2) immediately after HA injection; 3) 7 days after the HA injection; 4) 30 days after the HA injection; 5) 60 days after the HA injection. The proposed study regimen allowed to assess the baseline condition of the examined joint before treatment was applied. An orthopedic specialist then performed injections of Biolevox™ HA ONE on an outpatient procedure in the treatment room. Intra-articular injections were performed from a lateral mid-patellar approach, with the knee bent at 90°. Performing a second assessment was meant to provide data of supplementation effect immediately after injection. The examination one week after the HA injection administration was intended to allow the elimination of any negative pain factors associated with the injection itself, while allowing early assessment of the vibroacoustic changes that would occur in the joint. Subsequent examinations one and two months after the injection were to assess the persistence or disappearance of these changes. In addition, before the HA administration and 7, 30, and 60 days after the injection, the Western Ontario and McMaster Universities Osteoarthritis Index (WOMAC) questionnaire was completed to assess the patient's functional status and monitor the progress of the treatment. Moreover, the control group was included during the research. The group was tested with accelometric sensors once, which allows to present the VAG results of the OA non-affected population.

The following VAG descriptors were measured in the study, reflecting the level of vibration generated during movement:

- VMS - variance of mean squares in 5 millisecond time windows - describes the variability of the signal waveform
- R4 - the average of the four minimum and maximum values of the signal - describes the amplitude
- P1 - the sum of the power spectral densities of the short-time Fourier transform in the frequency intervals 50-250 Hz - expresses the proportion of oscillations with frequencies in a given interval
- P2 - sum of power spectral densities of short-time Fourier transform in frequency intervals 250-450 Hz - expresses the proportion of oscillations with frequencies in a given interval

The signal was recorded in the frequency range of 0.7-1000 Hz, with a sampling rate of 10 kHz. The obtained data were subjected to high-pass filtering with a cutoff level of 50 Hz to suppress noise (e.g., muscle tremors), and then analyzed.

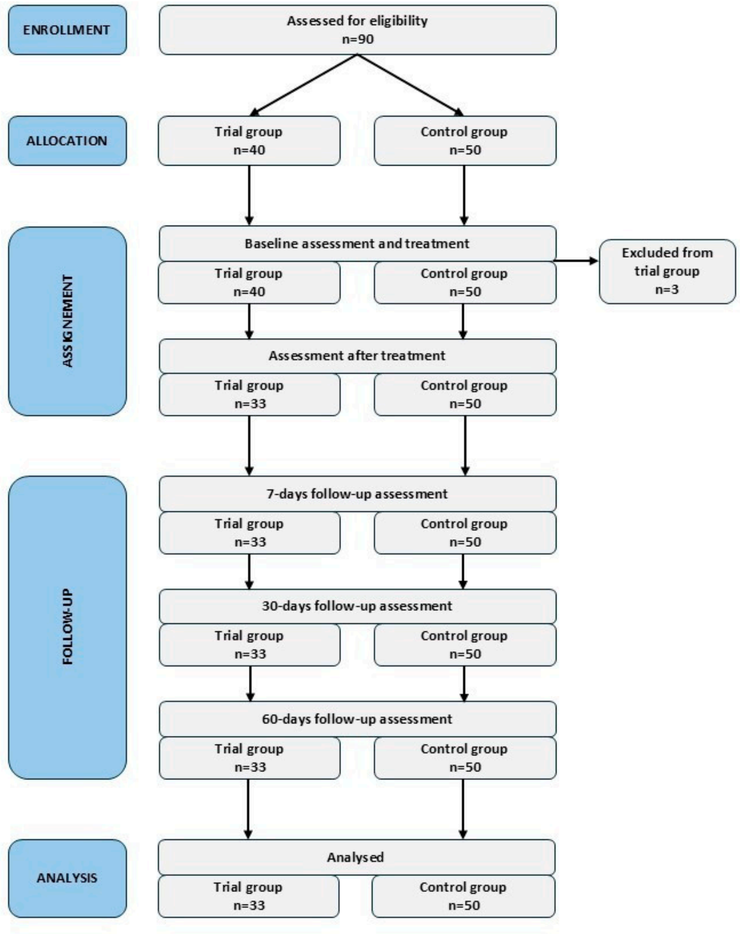

Figure S1. Flow-chart of patient selection during the study.
